# Effectiveness of BNT162b2 mRNA COVID-19 vaccine against SARS-CoV-2 variant Beta (B.1.351) among persons identified through contact tracing in Israel: A prospective cohort study

**DOI:** 10.1016/j.eclinm.2021.101190

**Published:** 2021-11-29

**Authors:** Shepherd R. Singer, Frederick J. Angulo, David L. Swerdlow, John M. McLaughlin, Itay Hazan, Netanel Ginish, Emilia Anis, Ella Mendelson, Orna Mor, Neta S Zuckerman, Oran Erster, Jo Southern, Kaijie Pan, Gabriel Mircus, Marc Lipsitch, Eric J. Haas, Luis Jodar, Yeheskel Levy, Sharon Alroy-Preis

**Affiliations:** a Public Health Services, Israel Ministry of Health, Jerusalem, Israel; b Hadassah Braun School of Public Health, Faculty of Medicine, Hebrew University, Jerusalem, Israel; c Pfizer Inc., Collegeville, PA, USA; d Ministry of Defense, Tel Aviv, Israel; e Central Virology Laboratory, Sheba Medical Center, Ramat Gan, Israel; f School of Public Health, Sackler Faculty of Medicine, Tel Aviv University, Tel Aviv, Israel; g Pfizer Pharmaceuticals Israel Ltd, Herzliya, Israel; h Harvard University, Boston, MA, USA; i Faculty of Health Sciences, Ben Gurion University of the Negev, Beer Sheva, Israel; j Israel Ministry of Health, Jerusalem, Israel

**Keywords:** COVID-19, SARS-CoV-2, vaccine effectiveness, prevention, mRNA vaccines, variant Beta (B.1.351), epidemiology

## Abstract

**Background:**

SARS-CoV-2 variant Beta (B.1.351) was designated as a Variant of Concern (VoC) after becoming the dominant strain in South Africa and spreading internationally. BNT162b2 showed lower levels of neutralizing antibodies against Beta than against other strains raising concerns about effectiveness of vaccines against infections caused by Beta. We estimated BNT162b2 vaccine effectiveness (VE) against Beta infections in Israel, a country with high vaccine uptake.

**Methods:**

The Ministry of Health (MoH) identified Beta cases through mandatory reporting of SARS-CoV-2 cases and whole genome sequencing (WGS) of specimens from vaccination-breakthrough infections, reinfections, arriving international travelers, and a selection of other infected persons. A cohort analysis was conducted of exposure events of contacts of primary Beta cases. WGS was conducted on available PCR-positive specimens collected from contacts. VE estimates with 95% confidence intervals (CIs) against confirmed and probable Beta infections were determined by comparing infection risk between unvaccinated and fully-vaccinated (≥7 days after the second dose) contacts, and between unvaccinated and partially-vaccinated (<7 days after the second dose) contacts.

**Findings:**

MoH identified 310 Beta cases through Jun 27, 2021. During the study period (Dec 11, 2020 – Mar 25, 2021), 164 non-institutionalized primary Beta cases, with 552 contacts aged ≥16 years, were identified. 343/552 (62%) contacts were interviewed and tested. 71/343 (21%) contacts were PCR-positive. WGS was performed on 7/71 (10%) PCR-positive specimens; all were Beta. Among SARS-CoV-2-infected contacts, 48/71 (68%) were symptomatic, 10/71 (14%) hospitalized, and 2/71 (3%) died. Fully-vaccinated VE against confirmed or probable Beta infections was 72% (95% CI -5 – 97%; p=0·04) and against symptomatic confirmed or probable Beta infections was 100% (95% CI 19 – 100%; p=0·01). There was no evidence of protection in partially-vaccinated contacts.

**Interpretation:**

In a prospective observational study, two doses of BNT162b2 were effective against confirmed and probable Beta infections. Through the end of June 2021, introductions of Beta did not interrupt control of the pandemic in Israel.

**Funding:**

Israel Ministry of Health and Pfizer.

**Research in context**

**Evidence before this study**

In a previously published study, the Israel MoH found BNT162b2 to be highly effective (>95%) in preventing SARS-CoV-2 infections, COVID-19, hospitalizations, and deaths during a period when the Alpha (B.1.1.7), was the dominant strain. SARS-CoV-2 variant Beta caused a surge of infections, rapidly became the dominant strain in South Africa, and was designated as a Variant of Concern (VoC). The international spread of Beta has increased the need for further data on the real-world effectiveness of BNT162b2 to prevent SARS-CoV-2 infections caused by this strain.

**Added value of this study**

Despite the multiple introductions of Beta into Israel, there was a low incidence of SARS-CoV-2 infections through Jun 27, 2021. In a cohort study of exposure events of contacts of primary Beta cases, two doses of BNT162b2 were effective in preventing confirmed and probable SARS-CoV-2 Beta infections and disease. One dose of BNT162b2 did not appear effective in preventing Beta infections.

**Implications of all the available evidence**

The sustained low incidence in Israel through the end of June 2021 despite multiple introductions of Beta and the apparent effectiveness in our observational study of two doses of BNT162b2 against infections caused by variant Beta indicates that in the absence of remarkable epidemiological changes the presence of the Beta variant in Israel is not likely to threaten the declines in SARS-CoV-2 infections, COVID-19 cases, hospitalizations, and deaths seen in Israel following the rapid achievement of high vaccine uptake.

Alt-text: Unlabelled box

## 1 Introduction

The SARS-CoV-2 pandemic resulted in >180M cases and >3.9M deaths worldwide through Jun 27, 2021 [1] including 840 888 cases and 6 428 deaths in Israel [2] (population 9·1M).

The Pfizer/BioNTech mRNA vaccine (BNT162b2), had 95% efficacy against symptomatic infections in a RCT [3]. Real-world studies have also demonstrated high VE against infections, [4,5] hospitalizations, and deaths [6,7]. Israel launched a vaccination campaign on Dec 20, 2020 that quickly achieved high vaccine coverage and resulted in sustained declines in the incidence of SARS-CoV-2 infections [8]. A Ministry of Health (MoH) study during a period when 95% of specimens were variant Alpha reported VEs of >95% against infections, symptomatic infections, hospitalizations, deaths in Israel [8]. The Israeli vaccination campaign is estimated to have averted more than 150 000 infections, 24 000 hospitalizations and 5500 deaths in Israel through Apr 10, 2021 [9].

Many SARS-CoV-2 variants have been identified [10]. Variant emergence requires frequent VE assessments as mutations may affect the protective effect of vaccination [10,11]. Beta possesses numerous mutations including the S gene resulting from amino acid substitutions which are thought to pose a potential risk to vaccine effectiveness [12]. Beta rapidly became the dominant strain in South Africa[12] and is classified by the World Health Organization (WHO) as a VoC due to increased transmissibility and virulence [13].

Laboratory studies have shown that sera from BNT162b2 recipients [14], [15], [16], [17], [18] and mRNA-1273 recipients [18,19] had less neutralizing activity against Beta than against Alpha or the vaccine strain WA-1. Non-mRNA-based vaccines NVX-CoV2372 and ChAdOx1 had efficacy of only 51% (95% CI -0·6 – 76·2%) and 21.9% (95% CI -49·9 – 59·8%), respectively, against Beta in RCTs among HIV-negative adults in South Africa [20,21]. By Jun 27, 2021, Beta was reported in 97 countries [22] with local transmission reported in Canada, Germany, the United Kingdom, and the United States [23]. Beta was first reported in Israel on Dec 29, 2020 [24]. An Israeli study reported that a higher proportion of vaccine breakthrough cases were Beta compared to other strains [25].

Given these concerns, VE estimates against Beta were needed. Estimating VE, however, requires a setting with sufficient disease and vaccine uptake in a jurisdiction with robust public health surveillance. The only previously available VE estimate against Beta is from Qatar which reported BNT162b2 effectiveness of 75% (95% CI 70·5 –78·9%) [26]. We therefore conducted a prospective cohort analysis to estimate VE against Beta in Israel.

## 2 Methods

### 2.1 Setting and population

MoH leads the ongoing Israel COVID-19 vaccination campaign. MoH conducts active surveillance for laboratory-confirmed SARS-CoV-2 infections, with mandatory daily reporting of PCR results by all diagnostic laboratories. MoH attempts to interview and obtain nasopharyngeal samples from all arriving international travelers. MoH conducts active surveillance of COVID-19-associated hospitalizations via daily updates received from all hospitals. Information about vaccine administration is also reported in real time to the national COVID-19 database.

MoH attempts to interview each person with a laboratory-confirmed SARS-CoV-2 infection. During the interview, the infected person is asked about symptoms and asked to provide the names of “contacts,” which are defined as individuals who resided in the infected person's household or who were within two meters of the infected person for at least 15 minutes during the “infectious period” of the infected person. For an infected person who was symptomatic, the infectious period is defined as 4 days before through 10 days after symptom onset. For an infected person who was asymptomatic, the infectious period is defined as 7 days before through 14 days after collection of the specimen that yielded the positive test. MoH attempts to obtain informed consent and interview contacts of infected persons for presence of symptoms and collect a nasopharyngeal swab for SARS-CoV-2 testing.

Surveillance of COVID-19, monitoring of vaccine uptake, and epidemiological investigations, are part of the national pandemic response and are performed under Public Health Ordinance 1940. Only de-identified data were utilized in this analysis. This study was approved by MoH's Institutional Review Board (CoR-MoH-080-2021). The study followed the Strengthening the Reporting of Observational studies in Epidemiology (STROBE) guidelines (see supplemental material for a completed STROBE checklist).

### 2.2 Testing for SARS-CoV-2 and variant Beta

SARS-CoV-2 testing is free-of-charge and widely available in Israel. Neither a physician referral nor presence of symptoms are required for testing. When seeking testing, individuals provide their identification number and a specimen is collected via a nasal or nasopharyngeal swab. Specimens are tested at clinical diagnostic laboratories using real-time PCR tests. Residual specimens from PCR-positive samples collected from patients considered at high risk of being infected with a SARS-CoV-2 variant were transferred to Israel's Central Virology Laboratory. These include specimens from suspected re-infections, vaccine breakthroughs, and returning international travelers. Additional PCR-positive samples were also selected and transferred to the central laboratory including all PCR-positive specimens starting in mid-April 2021. RNA from PCR-positive specimens are screened for specific spike gene mutations by PCR using the Seegene Novaplex SARS-CoV-2 variants I assay that detects RdRp (positive control) and the following changes in the spike protein: N501Y and HV 69/70 deletion indicative of Alpha, and E484K and N501Y indicative of Beta (Seegene Inc., Seocho-gu, Seoul, 06646, South Korea). The central laboratory recently developed a unique assay that specifically detects variant Beta [24]. WGS is then conducted at MoH qualified laboratories that participate in the national sequencing consortium.

WGS (see Supplemental references) use the COVID-seq library preparation kit (Illumina). Library validation and mean fragment size are determined by TapeStation 4200 via DNA HS D1000 kit (Agilent). Pooled libraries are denatured, diluted to 10pM, and sequenced on NovaSeq (Illumina). Resulting fastq files are subjected to quality control using FastQC and MultiQC. Low-quality sequences are filtered using Trimmomatic. Sequences are mapped to the SARS-CoV-2 reference genomes (NC_045512.2) using Burrows-Wheeler Aligner (BWA). Resulting binary alignment map files are sorted and indexed using the SAMtools suite. Consensus fasta sequences are assembled using iVar, with positions with <5 nucleotides determined as Ns. Multiple alignment of sample sequences with SARS-CoV-2 reference genome (NC_045512.2) are done with MAFFT. Alpha and Beta specific mutations are located via a custom Python script.

### 2.3 Design and definitions

A primary Beta case was defined as WGS-confirmed Beta case identified by MoH surveillance activities other than MoH contact tracing program. Beta cases identified by contact tracing were considered secondary Beta cases. We conducted a cohort analysis of exposure events of contacts aged ≥16 years of non-institutionalized primary Beta cases from Dec 11, 2020 – Mar 15, 2021 (only persons aged ≥16 years were eligible for vaccine during this period). The cohort analysis of the exposure events of the contacts included secondary Beta cases but did not include primary Beta cases. Primary Beta cases were not included in the cohort analysis because vaccine history contributed to the identification of some primary Beta cases. For the contacts of primary Beta cases, an exposure event for the cohort analysis was defined as an exposure by a contact to a primary Beta case during the infectious period of the primary case (4 days before to 10 days after symptom onset or, for a asymptomatic case, 7 days before to 14 days after the specimen collection date). Exposure events were included in the cohort analysis if the contact did not have a previous laboratory-confirmed SARS-CoV-2 infection. If a contact had multiple exposures to a primary Beta case during the infectious period of the case, only the earliest exposure event was included in the cohort analysis. Contacts who were exposed to additional infectious primary Beta cases beyond the infectious period of the initial case could contribute additional exposure events to the cohort analysis. However, once a contact was PCR-positive, any subsequent exposure event after the PCR-positive result was censored because the contact was no longer at risk of infection. Available PCR-positive specimens from contacts with exposure events in the cohort analysis were tested by WGS.

Vaccination history for a contact involved in an exposure event was determined at the earliest date that the contact was in contact with that infectious primary Beta case. At that date, the contact was defined as fully-vaccinated (received two BNT162b2 doses with ≥7 days after the second dose), partially-vaccinated (received only one dose at ≥14 days after the first dose or received two doses with <7 days after the second dose), initially-vaccinated (received only one dose <14 days after the first dose), or unvaccinated. Contacts of primary Beta cases who were SARS-CoV-2 infected were defined as being a “confirmed” Beta case if confirmed by WGS or “probable” Beta case if WGS was not performed.

Risks of having a SARS-CoV-2 infection, symptomatic disease, hospitalization, or death caused by Beta were calculated for the exposure events involving fully-vaccinated, partially-vaccinated, initially-vaccinated, and unvaccinated contacts of primary Beta cases. The Fisher's Exact Test was used to test the association of vaccine status and infection risk [27]. VE against Beta infections at ≥7 days after the second dose was estimated as one minus the risk ratio by comparing the infection risk of exposure events involving fully-vaccinated and unvaccinated contacts. Similarly, VE at <7 days after the second dose was estimated by comparing the infection risk of exposure events involving partially-vaccinated and unvaccinated contacts. Ninety-five percent confidence intervals (CIs) were calculated using the Clopper-Pearson method [27]. All analyses were conducted using SAS version 9.4

### 2.4 Role of the funding source

MoH and Pfizer separately provided in-kind support to this study. No funding was exchanged between the Israel MoH and Pfizer. MoH and Pfizer were involved in the study design and the writing of the report. MoH and Pfizer approved the decision to submit the manuscript for publication.

## 3 Results

**Figure 1:**
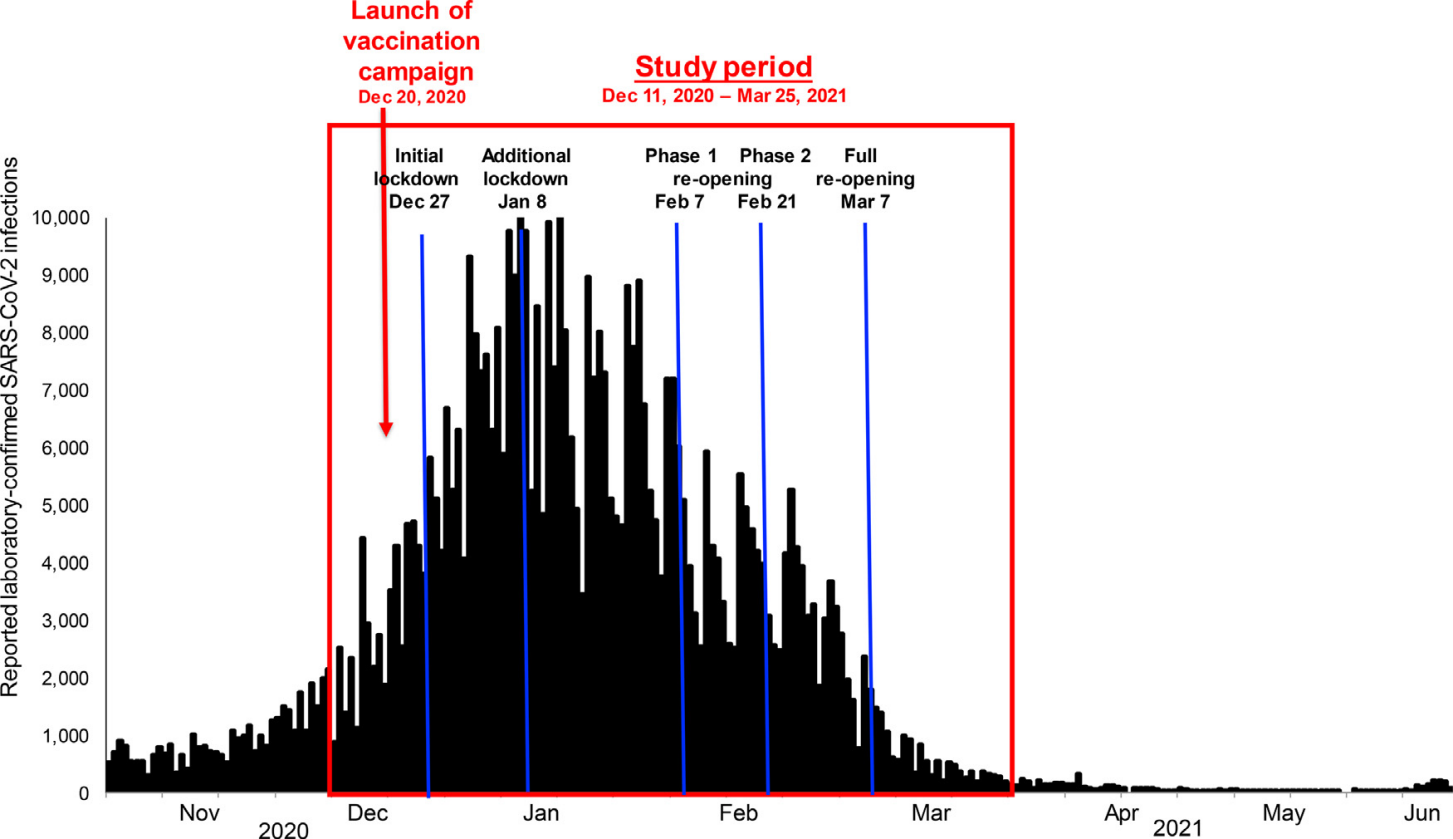
Daily reported laboratory-confirmed SARS-CoV-2 infections, Nov 1, 2020 – Jun 27, 2021, Israel

**Figure 2:**
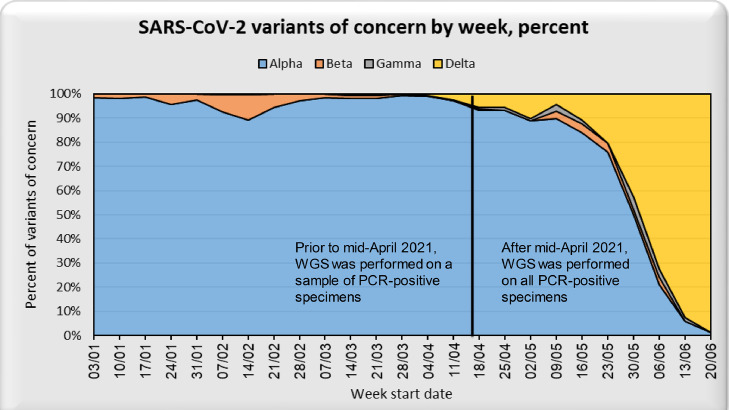
Percent of PCR-positive specimens that are SARS-CoV-2 variants of concern (VoC), by week, Jan 3, 2021 – Jun 27, 2021, Israel

The number of reported daily SARS-CoV-2 infections declined markedly in Israel beginning in Jan 2021 (Figure 1). Through Jun 27, 2021, 310 WGS-confirmed Beta cases were identified in Israel (Figure 2). While Beta cases did not increase in prevalence through June 2021, there was a dramatic increase in variant Delta (B.1.617.2) cases in June 2021.

**Figure 3:**
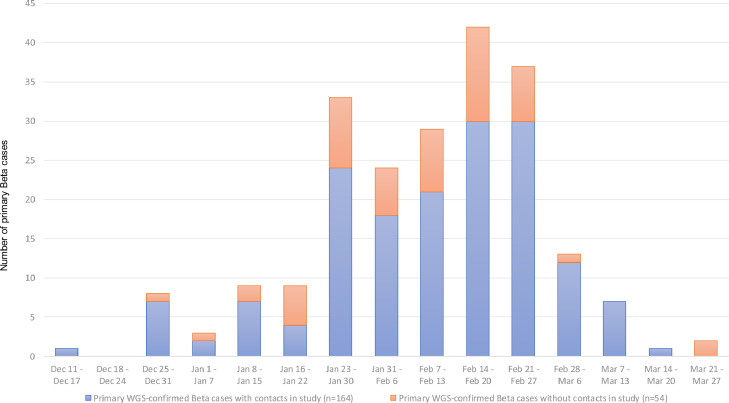
Number of primary whole genome sequencing (WGS)-confirmed Beta cases identified during study period, Dec 11, 2020 – Mar 25, 2021, with contacts in the study, by week of laboratory-confirmation of SARS-CoV-2 infection, Israel.

**Figure 4:**
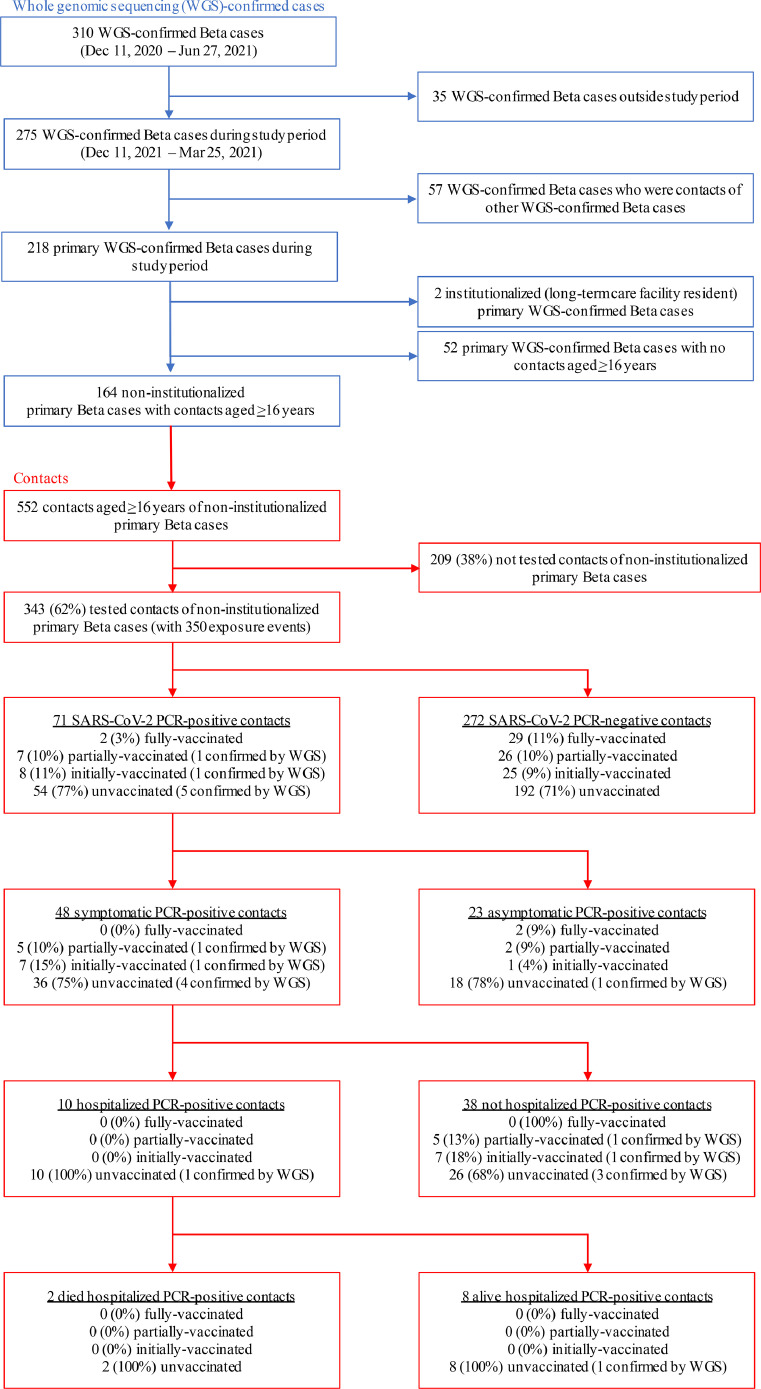
Contacts of primary Beta cases, Israel, Dec 11, 2020 – Mar 25, 2021.

During the study period (Dec 11, 2020 – Mar 15, 2021), 218 primary Beta cases who were not contacts of other primary Beta cases were identified (Figure 3, Figure 4). Two primary Beta cases who were residents of a long-term care facility were excluded. Fifty-two primary Beta cases reported not having any contacts aged ≥16 years during their infectious period. The remaining 164 non-institutionalized primary Beta cases named 552 contacts aged ≥16 years. Of the 552 contacts, 343 (62%) were interviewed and had a specimen collected for SARS-CoV-2 testing. None of the contacts reported a SARS-CoV-2 infection prior to the interview. The median age of the 343 tested contacts was 40 years (range 16 – 94 years); 190 (55%) were female. The 343 contacts had 350 exposure events with an infectious primary Beta case (7 contacts were exposed to an additional infectious primary Beta case beyond the infectious period of the initial case).

PCR=polymerase chain reaction. WGS=whole genome sequencing.

Of the tested contacts, 31/343 (9%) were fully-vaccinated, 32/343 (9%) were partially-vaccinated, 34/343 (10%) were initially-vaccinated, and 246/343 (72%) were unvaccinated at the date of first exposure to an infectious primary Beta case (Table 1). Of tested contacts, 71/343 (21%) were PCR positive (Table 2). The 7 contacts with exposure to two primary Beta cases were PCR negative following both exposures. Of the SARS-CoV-2 infected contacts, 2/71 (3%) were fully-vaccinated, 7/71 (10%) were partially-vaccinated, 8/71 (11%) were initially-vaccinated, and 54/71 (76%) were unvaccinated.

**Table 1:** Age, sex, and sector of contacts of non-institutionalized primary Beta cases by vaccination status,* Israel, Dec 11, 2020 – Mar 25, 2021. (N=343^)

	Fully-Vaccinated (n=31)	Partially-vaccinated (n=32)	Initially-Vaccinated(n=34)	Unvaccinated (n=246)	Total (n=343)
**Age (median, range)**					
Years	58 (21 – 87)	38 (18 – 72)	40 (16 –57)	39 (16 – 94)	40 (16 – 94)
					
**Sex (n, percent)**					
Male	14 (45)	12 (37)	19 (56)	108 (44)	153 (45)
Female	17 (55)	20 (62)	15 (44)	138 (56)	190 (55)
					
**Sector (n, percent)**					
Arab	8 (26)	13 (41)	13 (38)	74 (30)	108 (31)
Ultra-orthodox	1 (3)	1 (3)	0 (0)	4 (2)	6 (2)
General Jewish (non-ultra-orthodox)	22 (71)	18 (47)	21 (62)	168 (68)	229 (67)

* Vaccination status: Fully-vaccinated = received two doses of BNT162b2 ≥7 days after the second dose. Initially-vaccinated = received one dose of BNT162b2 <14 days after the first dose. Partially-vaccinated = received one dose of BNT162b2 ≥14 days after the first dose or two doses <7 days after the second dose. Unvaccinated = never received BNT162b2.

^Seven contacts were exposed to an additional infectious primary Beta case, resulting in 350 exposure events in the cohort analysis. The median age of the seven contacts with exposure to two cases was 31 years (range 26-87); 1/7 (14%) were male. The vaccination history was 2/7 (28%) fully-vaccinated, 3 (43%) initially-vaccinated, 0 partially-vaccinated, and 2 (28%) unvaccinated

**Table 2:** Age, sex, and sector of contacts of non-institutionalized primary Beta cases by SARS-CoV-2 PCR result, Israel, Dec 11, 2020 – Mar 25, 2021. (N=343^)

	SARS-CoV-2 PCR positive: confirmed or probable Beta infection (n=71)	SARS-CoV-2 PCR negative: uninfected (n=272)
**Age (mean, range)**		
Years	35 (16 – 89)	42 (16 –94)
		
**Sex (n, percent)**		
Male	36 (51)	117 (43)
Female	35 (49)	155 (57)
		
**Sector (n, percent)**		
Arab	28 (39)	80 (29)
Ultra-orthodox	0 (0)	6 (2)
General Jewish (non-ultra-orthodox)	43 (61)	186 (68)

^Seven contacts were exposed to an additional infectious primary Beta cases, resulting in 350 exposure events in the cohort analysis. All seven contacts were PCR negative following the exposure events with both cases.

A specimen was available for additional testing by WGS from 7/71 (10%) PCR-positive contacts of WGS-confirmed Beta cases that had exposure events in the cohort analysis. All 7 specimens were confirmed as SARS-CoV-2 variant Beta by WGS. Of the contacts with confirmed Beta infection, none were fully-vaccinated, 1/7 (14%) was partially-vaccinated, 1/7 (14%) was initially-vaccinated, and 5/7 (71%) were unvaccinated. Among the 71 SARS-CoV-2 infected contacts (7 with confirmed and 64 with probable Beta infections), 48/71 (68%) had symptoms, 10/71 (14%) were hospitalized, and 2/71 (4%) died. Among contacts with symptomatic confirmed or probable Beta infections, 0/48 were fully-vaccinated, 5/48 (10%) were partially-vaccinated, 7/48 (14%) were initially-vaccinated, and 36/48 (75%) were unvaccinated. Among the 10 hospitalized contacts infected with Beta, all were unvaccinated. The two contacts infected with Beta that died were unvaccinated.

In the cohort analysis of 350 exposure events of tested contacts of non-institutionalized primary Beta cases, the risk of the contact becoming a confirmed or probable Beta case was 6% (2/33) among exposure events of fully-vaccinated contacts, 21% (7/33) among exposure events of partially-vaccinated contacts, 22% (8/36) among exposure events of initially-vaccinated contacts, and 22% (54/248) among exposure events of unvaccinated contacts (Table 3). The VE against Beta infection was 72% (95% CI -5 – 97%; p= 0·04) at ≥7 days after the second dose. The risk of the contact becoming a symptomatic confirmed or probable Beta case was 0% (0/33) among exposure events of fully-vaccinated contacts, 15% (5/33) among exposure events of partially-vaccinated contacts, 19% (7/36) among exposure events of initially-vaccinated contacts, and 14% (36/248) among exposure events of unvaccinated contacts. The VE against symptomatic Beta infection was 100% (95% CI 19 – 100%; p=0·01) at ≥7 days after the second dose. None of the exposure events of fully-vaccinated, partially-vaccinated, or initially-vaccinated contacts resulted in a hospitalized Beta infection but 4% (10/248) of the exposure events of unvaccinated contacts resulted in a hospitalized Beta infection. There was no evidence of effectiveness of being partially-vaccinated against Beta infection or against symptomatic Beta infection; i.e., there was no statistical difference (p>0·99) between the infection risk between the exposure events of partially vaccinated contacts and unvaccinated contacts for either outcome.

**Table 3:** Infection risk and vaccine effectiveness against confirmed or probable* Beta infection, by vaccination status,**in cohort of exposure events by contacts of non-institutionalized primary Beta cases, Israel, Dec 11, 2020 – Mar 25, 2021. (N=350)

	n/n (%)	VE (95% CI; p value)***
Infection		
Unvaccinated	54/248 (22)	Ref
Initially-vaccinated	8/36 (22)	—-
Partially-vaccinated	7/33 (21)	3 (-115 – 63; p >0·99)
Fully-vaccinated	2/33 (6)	72 (-5 – 97; p = 0·04)
Total	71/350 (20)	
**Symptomatic infection**		
Unvaccinated	36/248 (14)	Ref
Initially-vaccinated	7/36 (19)	—-
Partially-vaccinated	5/33 (15)	-4 (-167 – 68; p >0·99)
Fully-vaccinated	0/33 (0)	100 (19 – 100; p = 0·01
Total	48/350 (14)	
**Hospitalization**		
Unvaccinated	10/248 (4)	Ref
Initially-vaccinated	0/36 (0)	—-
Partially-vaccinated	0/33 (0)	100 (-235 – 100; p = 0·61)
Fully-vaccinated	0/33 (0)	100 (-235 – 100; p = 0·61)
Total	10/350 (3)	
**Death**		
Unvaccinated	2/248 (0.8)	Ref
Initially-vaccinated	0/36 (0)	—-
Partially-vaccinated	0/33 (0)	100^
Fully-vaccinated	0/33 (0)	100^
Total	2/350 (0.6)	

VE = vaccine effectiveness; CI = confidence interval; Ref = reference group for VE calculation.

*Probable Beta infection is a SARS-CoV-2 PCR positive contact of a non-institutionalized primary Beta case without whole genome sequencing results.

**Vaccination status: Unvaccinated = never received BNT162b2. Initially-vaccinated = received one dose of BNT162b2 <14 days after the first dose. Partially-vaccinated = received one dose of BNT162b2 ≥14 days after the first dose or two doses <7 days after the second dose. Fully-vaccinated = received two doses of BNT162b2 ≥7 days after the second dose.

***95% CIs were calculated using the Clopper Pearson method and p values were determined using Fisher's Exact Test.

^CIs and p value not given if total number of cases <10 for a given infection type.

## 4 Discussion

In our analysis, BNT162b2 was effective against symptomatic confirmed and probable Beta infections and against all (asymptomatic and symptomatic) Beta infections at ≥7 days after the second dose. The VE estimate against symptomatic Beta infections in our study is comparable to the VE estimate (97%) against symptomatic Alpha infections by the MoH [8]. A comparable VE estimate against all (asymptomatic and symptomatic) Beta infections (75%) as the estimate in our study has recently been reported by Qatar, [26] but our study is the first to report VE against Beta confirmed by WGS. The VE estimate against all Beta infections in our study is also comparable to the VE estimate against all SARS-CoV-2 infections (65%) in Israel by Regev-Yochay et al., that, like our study, used a contact-tracing design (i.e., history of exposure to an infected case) to ascertain infected persons [28]. A contract-tracing study design is likely to result in ascertainment of a higher proportion of asymptomatic or milder infections, and thus a lower VE estimate against all infections compared to an approach which does not actively trace contacts of cases[29]. For example, an MoH study which included all infections nationwide rather than a contact-tracing study design, estimated a higher VE against Alpha infections (95%) [8] than the contemporaneous Regev-Yochay study [28].

The high VE against symptomatic Beta infections in our study is comparable to the 95% efficacy of BNT162b2 against symptomatic SARS-CoV-2 infections in the initial report of the RCT which included infections predominately from the United States from Jul - Nov 2020 [3]. A follow-up report of the RCT reported that all nine symptomatic SARS-CoV-2 cases among the 800 RCT participants in South Africa that occurred between Dec 17, 2020 and Feb 2, 2021 were in the placebo group for a vaccine efficacy of 100% (95% CI 53·5 – 100%) [30]. The SARS-CoV-2 strains from these nine cases were sequenced and eight were variant Beta, supporting high effectiveness against Beta despite concerns of lower levels of neutralizing antibody against Beta compared to other strains [30].

Our results emphasize the importance of measuring VE in a real-world study in addition to studies on immunological parameters (e.g. neutralizing antibody titers) when considering the effectiveness of vaccines against emerging variants or the need for administering a booster dose [31]. Several studies have reported that sera from vaccine recipients had less neutralizing activity against Beta than against other strains [14], [15], [16], [17], [18], [19]. For example, when performing plaque-reduction neutralization testing using sera from RCT BNT162b2-recipients at two to four weeks after the second dose, neutralization titers of Beta-spike virus were lower than titers to other strains [14]. The results from the present study, supported by data from Qatar [26] and the recent additional RCT analysis of South African participants,[30] demonstrates real-world protection against SARS-CoV-2 infections caused by Beta, and provide reassurance that the BNT162b2 vaccine can be used effectively in settings where Beta is circulating.

Our study also highlights the importance of administering two doses of BNT162b2. We found no evidence that partially-vaccinated individuals had protection against Beta. These results are consistent with the study in Qatar which estimated low VE (17%) of one dose of BNT162b2 against presumed Beta infections [26]. Taken together, these findings reinforce the need for a two-dose schedule of BNT162b2 for adequate protection against Beta infection and disease.

Israel presented a unique opportunity to assess VE against Beta. MoH conducts epidemiologic investigations of every reported SARS-CoV-2 case with tracing and testing of contacts. We used a cohort analysis of exposure events of contacts and excluded the primary Beta cases from the analysis. The primary Beta cases were excluded because the vaccine history of some primary Beta cases may have influenced their identification as cases (i.e., some primary Beta cases were identified because they were vaccine breakthrough cases or re-infections) and some primary Beta cases may have been less likely to be vaccinated (i.e., travelers returning from abroad). We therefore conducted the VE estimation exclusively using exposure events of contacts of primary Beta cases to avoid the potential selection bias associated with identification of the primary Beta cases. We also excluded two primary Beta cases who were long-term care facility residents because it difficult to distinguish among the cases in an institutional setting as to which cases are primary cases and which are secondary cases. This unique study design of using a cohort of exposure events of non-institutionalized contacts could be applied in the future assessments of other SARS-CoV-2 variants.

Our investigation utilized several approaches to minimize misclassification. We reduced inaccuracies in vaccine status in our study by using Israel's national database of vaccine administrations. However, the date of earliest contact between an infectious primary Beta care and the contact, which is the date used for classifying vaccination status for the contact involved in the exposure event in our cohort analysis, relied on the recall of the primary Beta case which may be subject to bias. While misclassification of an unvaccinated contact as vaccinated is unlikely, misclassification of vaccination status is possible (incorrect classification of fully-vaccinated, partially-vaccinated, or initially-vaccinated) if the date of earliest contact between the primary Beta case and contact is inaccurate. We judge, however, that this misclassification is not a major problem in our study since it is likely to be nondifferential with respect to disease outcomes (making it more difficult to demonstrate an effect) and given the clear distinction of VE estimates for fully-vaccinated and partially-vaccinated individuals in our study.

We identified Beta cases using WGS, the gold standard for identifying SARS-CoV-2 variants. All of the primary Beta cases were confirmed by WGS but only 10% of the Beta cases in our cohort analysis (i.e., the secondary Beta case) were confirmed by WGS. It can be reasonably assumed that the SARS-CoV-2-infected contacts who did not have a specimen additional sequenced had a Beta infection because they had close contacts with a WGS-confirmed Beta-infected persons during the time when the Beta-infected person was infectious. However, the fact that all tested specimens from contacts in the cohort analysis were confirmed to be Beta adds confidence that most of the probable Beta infections in our cohort analysis are likely to be Beta infections.

Another limitation of our study is that, although we included all tested contacts of non-institutionalized primary Beta cases in Israel in our study, the relative sample size of our cohort remained small. There was sufficient power to evaluate estimates of VE against SARS-Cov-2 infections caused by Beta and VE against symptomatic infections caused by Beta but the confidence intervals are wide and there was insufficient power to assess VE against hospitalizations or deaths caused by Beta. Additional studies are needed to assess VE against these outcomes. Furthermore, there was insufficient power to adjust for covariates or to adjust for clustering of multiple exposure.

Beta was first identified in Israel in late Dec 2020. Through Jun 27, 2021, 310 WGS-confirmed Beta cases were identified in Israel. However, the multiple introductions of Beta into Israel were not associated with an increase in the daily number of reported SARS-CoV-2 cases. Instead, despite complete societal reopening, there was a low incidence of SARS-CoV-2 infections in Israel following the high vaccine uptake of BNT162b2. Taken together with the VE against Beta reported in this study, these data indicate that the high-vaccine uptake of two doses of BNT162b2 in Israel contributed to the control the pandemic despite the multiple introductions of Beta. There has been, however, a recent dramatic increase in Delta infections in Israel since June and there now is an urgent need for VE estimates against Delta infections.

As variants emerge, particularly those with novel genetic mutations that affect the function of the SARS-CoV-2 virus, further real-world VE studies will be required to evaluate the sustained protection provided by COVID-19 vaccines, to inform the need for booster doses, and to develop next generation vaccines. Studying VE among a cohort of contacts, the methodology described in this report, may be useful in other epidemiological circumstances when it is appropriate to exclude primary cases (i.e., when identification of cases may have been influenced by vaccine history). Given the concerns about the potential reduced VE of COVID-19 vaccines against Beta and the international dissemination of Beta, this study provides real-world evidence that two doses of BNT162b2 is likely to provide protection against SARS-CoV-2 infections caused by Beta.

## Data sharing

Requests for data should be made to the Ministry of Health of Israel. Aggregated surveillance data are freely available online at https://data.gov.il/dataset/covid-19.

## Contributors

SS, FA, DS, JM, LJ, and SAP conceived this study, SS and SAP conducted the analysis and edited the final manuscript. SS, JS, FA, JM, and DS wrote the first draft of the protocol. SS, IH, and NG had access to the raw data. SS, JM, FK, and KP cleaned and analyzed the data. IH and NG performed the data analysis and edited the final manuscript. EM, OM, NZ and OE performed the laboratory analysis and edited the final manuscript. All authors contributed to the study design. All authors contributed to drafting the protocol and revised the manuscript for important intellectual content. All authors gave final approval of the version to be published.

## Funding

The Israel Ministry of Health and Pfizer separately provided in-kind support to this study. No funding was exchanged between the Israel Ministry of Health and Pfizer. No individual-level or sensitive data were exchanged between parties.

## Declaration of Competing Interest

Frederick Angulo, David Swerdlow, John McLaughlin, Farid Khan, Gabriel Mircus, Kaijie Pan, Jo Southern, and Luis Jodar are employees of Pfizer Inc, and hold stock and stock options in Pfizer Inc. Marc Lipsitch has provided advice on COVID-19 free of charge to Janssen, Astra-Zeneca, Pfizer, and COVAXX (United Biomedical), as well as to the nonprofit One Day Sooner and has received consulting income or honoraria from Merck, Pfizer, Bristol Meyers Squibb, Janssen, and Sanofi, and institutional research support from Pfizer. He is on the Scientific Advisory Committee of the Coalition for Epidemic Preparedness and Innovations (CEPI). All other authors report no conflicts.

## Appendix Supplementary materials

Image, application 1

## References

[1] Worldometers COVID-19 Coronavirus Pandemic. https://www.worldometers.info/coronavirus (accessed Aug 9, 2021 ).

[2] (Aug 9, 2021). Israel Ministry of Health website. (in Hebrew).

[3] Polack Polack FP, Thomas SJ, Kitchin N (Dec, 2020). Safety and efficacy of the BNT162b2 mRNA Covid-19 vaccine. New England Journal of Medicine 2020.

[4] Hall VJ, Foulkes S, Saei A (Apr 23, 2021). COVID-19 vaccine coverage in health-care workers in England and effectiveness of BNT162b2 mRNA vaccine against infection (SIREN): a prospective, multicentre, cohort study. Lancet 2021.

[5] Moustsen-Helms IR, Emborg HD, Nielsen J (Aug 9, 2021). Vaccine effectiveness after 1st and 2nd dose of the BNT162b2 mRNA Covid-19 vaccine in long-term care facility residents and healthcare workers – a Danish cohort study. MedRxiv preprint server..

[6] Lopez Bernal J, Andrews N, Gower C, Robertson C, Stowe J, Tessier E, Simmons R, Cottrell S, Roberts R, O'Doherty M, Brown K, Cameron C, Stockton D, McMenamin J, Ramsay M (May 13, 2021). Effectiveness of the Pfizer-BioNTech and Oxford-AstraZeneca vaccines on covid-19 related symptoms, hospital admissions, and mortality in older adults in England: test negative case-control study. BMJ 2021.

[7] Cavanaugh AM, Fortier S, Lewis P (March 2021). COVID-19 outbreak associated with a SARS-CoV-2 R.1 lineage variant in a skilled nursing facility after vaccination program — Kentucky. Morbidity and Mortality Weekly Report 2021.

[8] Haas EJ, Angulo FJ, McLaughlin JM (May 5, 2021). Impact and effectiveness of mRNA BNT162b2 vaccine against SARS-CoV-2 infections and COVID-19 cases, hospitalisations, and deaths following a nationwide vaccination campaign in Israel: an observational study using national surveillance data. Lancet 2021.

[9] Haas EJ, McLaughlin JM, Khan F (Sep 22, 2021). Infections, hospitalizations, and deaths averted via direct effects of the Pfizer-BioNTech BNT162b2 mRNA COVID-19 vaccine in a nationwide vaccination campaign, Israel. Lancet Infectious Diseases 2021.

[10] Lauring AS, Hodcraft EB. (2021). Genetic variants of SARS-CoV-2—what do they mean?. JAMA.

[11] Karim SS, Oliviera T. (March 24, 2021). New SARS-CoV-2 variants — Clinical, public health, and vaccine implications. New England Journal of Medicine 2021.

[12] Tegally H, Wilkinson E, Giovanetti M (Jul 9, 2021). Emergence and rapid spread of a new severe acute respiratory syndrome-related coronavirus 2 (SARS-CoV-2) lineage with multiple spike mutations in South Africa. MedRxiv preprint server.

[13] World Health Organization. Tracking SARS-CoV-2 variants. https://www.who.int/en/activities/tracking-SARS-CoV-2-variants/(accessed Aug 9, 2021).

[14] Kuzmina A, Khalaila Y, Voloshin O (2021). SARS CoV-2 spike variants exhibit differential infectivity and neutralization resistance to convalescent or post-vaccination sera. Cell Host and Microbe.

[15] Liu Y, Liu J, Xia H (Feb 17. 2021). Neutralizing activity of BNT162b2-elicited serum - Preliminary report. New England Journal of Medicine 2021.

[16] Skelly DT, Harding AC, Gilbert-Jaramillo J (Aug 9, 2021). Two doses of SARS-CoV-2 vaccination induce more robust immune responses to emerging SARS-CoV-2 variants of concern than does natural infection. Research square preprint server.

[17] Wang Z., Schmidt F., Weisblum Y. (2021). mRNA vaccine-elicited antibodies to SARS-CoV-2 and circulating variants. Nature.

[18] Lustig Y, Nemet I, Kliker L (Apr 7, 2021). Neutralizing response against variants after SARS-CoV-2 infection and one dose of BNT162b2. New England Journal of Medicine 2021.

[19] Wu K, Werner AP, Koch M (2021). Serum neutralizing activity elicited by mRNA-1273 vaccine. New England Journal of Medicine.

[20] Shinde V, Bhikha S, Hoosain X (May 5, 2021). Efficacy of NVX-CoV2373 Covid-19 vaccine against the B.1.351 variant. New England Journal of Medicine 2021.

[21] Madhi SA, Baillie V, Cutland CL (Mar 16, 2021). Efficacy of the ChAdOx1 nCoV-19 Covid-19 vaccine against the B.1.351 variant. New England Journal of Medicine 2021.

[22] (Aug 9, 2021). Global report investigating novel coronavirus haplotypes (B.1.351).

[23] Feder KA, Pearlowitz M, Goode A (January–February 2021). Linked clusters of SARS-CoV-2 variant B.1.351 — Maryland. Morbidity and Mortality Weekly Report 2021.

[24] Erster O, Mendelson E, Levy V (Aug 9, 2021). Rapid And high throughput RT-qPCR assay for identification and differentiation between SARS-CoV-2 variants B.1.1.7 and B.1.351. MedRxiv preprint server.

[25] Kustin T, Harel N, Finkel U (June 14, 2021). Evidence for increased breakthrough rates of SARS-CoV-2 variants of concern in BNT162b2-mRNA-vaccinated individuals. Nature Medicine.

[26] Abu‑Raddad LJ, Chemaitelly H, Butt AA (May 5, 2021). Effectiveness of the BNT162b2 Covid-19 vaccine against the B.1.1.7 and B.1.351 variants. New England Journal of Medicine 2021.

[27] Agresti A., Agresti A (2002). Categorical data analysis.

[28] Regev-Yochay G, Amit S, Bergwerk M (Aug 9, 2021). Decreased infectivity following BNT162b2 vaccination. SSRN preprint server.

[29] Lipsitch M, Kahn R. (Jun 23, 2021). Interpreting vaccine efficacy trial results for infection and transmission. Vaccine 2021.

[30] Thomas SJ, Edson M, Kitchin N (Aug 9, 2021). Six Month Safety and efficacy of the BNT162b2 mRNA COVID-19 vaccine. MedRxiv preprint server.

[31] Rubin R. (2021). COVID-19 Vaccines vs Variants—Determining How Much Immunity Is Enough. JAMA.

