# Supplementary material for: Effectiveness of BNT162b2 mRNA COVID-19 vaccine against SARS-CoV-2 variant Beta (B.1.351) among persons identified through contact tracing in Israel: A prospective cohort study
Source: eClinicalMedicine. 2021 Nov 29;42:101190. doi: 10.1016/j.eclinm.2021.101190 (PMC8628463; doi:10.1016/j.eclinm.2021.101190)
Supplement: Supplementary file 1 [file mmc1.docx]

Supplemental material for “Effectiveness of BNT162b2 mRNA COVID-19 vaccine against SARS-CoV-2 variant Beta (B.1.351) in Israel”

Additional references for whole genome sequencing

1. Babraham Bioninformatics. FastQC: A quality control tool for high throughput sequence data. <https://www.bioinformatics.babraham.ac.uk/projects/fastqc/> (accessed Jun 22, 2021).
2. Ewels P, Magnusson M, Lundin S, Käller M. MultiQC: summarize analysis results for multiple tools and samples in a single report. *Bioinformatic*s 2016;32(19):3047–3048. <https://www/doi.org/10.1093/bioinformatics/btw354>
3. Bolger AM, Lohse M, Usadel B. Trimmomatic: a flexible trimmer for Illumina sequence data, *Bioinformatics* 2014;30 (15):2114–2120. <https://www.doi.org/10.1093/bioinformatics/btu170>
4. Li H, Durbin R. Fast and accurate long-read alignment with Burrows–Wheeler transform. *Bioinformatics* 2010;26(5):589–595. <https://www.doi.org/10.1093/bioinformatics/btp698>
5. Li H. A statistical framework for SNP calling, mutation discovery, association mapping and population genetical parameter estimation from sequencing data. *Bioinformatics* 2011;27(21):2987–2993. <https://www.doi.org/10.1093/bioinformatics/btr509>
6. Grubaugh ND, Gangavarapu K, Quick J, et al. An amplicon-based sequencing framework for accurately measuring intrahost virus diversity using PrimalSeq and iVar. *Genome Biol* 2019;20(8). <https://www.doi.org/10.1186/s13059-018-1618-7>
7. Katoh K, Misawa K, Kuma K, Miyata T. MAFFT: a novel method for rapid multiple sequence alignment based on fast Fourier transform. *Nucleic Acids Research* 2002;30(14):3059–3066. <https://www.doi.org/10.1093/nar/gkf436>
8. Tang JW, Tambyah PA, Hui DS. Emergence of a new SARS-CoV-2 variant in the UK. *J Infect* 2021;82(4):e27-e28. <https://www.doi.org/10.1016/j.jinf.2020.12.024>.
